# Identification of microRNAs in bovine faeces and their potential as biomarkers of Johne’s Disease

**DOI:** 10.1038/s41598-020-62843-w

**Published:** 2020-04-03

**Authors:** Ronan G. Shaughnessy, Damien Farrell, Bojan Stojkovic, John A. Browne, Kevin Kenny, Stephen V. Gordon

**Affiliations:** 1 School of Veterinary Medicine, University College Dublin, Dublin 4, Ireland; 2 School of Medicine, University College Dublin, Dublin 4, Ireland; 3 School of Biomolecular and Biomedical Science, University College Dublin, Dublin 4, Ireland; 4 School of Agriculture and Food Science, University College Dublin, Dublin 4, Ireland; 5 UCD Conway Institute, University College Dublin, Dublin 4, Ireland; 6 Department of Agriculture, Food and the Marine, Central Veterinary Research Laboratory, Stacumny Lane, Backweston, Co. Kildare, Ireland; 7 Present Address: Biosensia, NovaUCD, Belfield Innovation Park, UCD, Dublin, Ireland; 8 Present Address: School of Biomedical Sciences and Pharmacy, University of Newcastle, New South Wales, Australia

**Keywords:** Infectious-disease diagnostics, Diagnostic markers

## Abstract

Extracellular microRNAs (miRNAs) are detectable in the peripheral blood and have been touted as potential biomarkers for a range of maladies. The presence and biomarker potential of miRNAs in other biofluids has been less thoroughly explored, particularly in the veterinary realm. Faecal miRNAs are a case in point; while they have been identified largely in rodents and humans, they have not been reported in cattle but may have prognostic or diagnostic value for Johne’s Disease (JD) in cattle, a chronic granulomatous inflammation of the ileum caused by *Mycobacterium avium* subspecies *paratuberculosis* (MAP). The aim of this study was thus to characterise the bovine faecal miRNome and to determine the utility of these transcripts as biomarkers for JD. Real-time PCR arrays consisting of 752 miRNA targets, optimised for detection of human miRNA, were used to screen RNA purified from faecal samples obtained from confirmed JD clinical cases vs. healthy controls. Two hundred and fifty-eight miRNAs were detected in bovine faeces, three of which are potentially novel orthologs of known human miRNAs. Differential abundance of three miRNA was evident in animals with clinical JD as compared to healthy controls. Our study has therefore identified a variety of miRNAs in bovine faeces and has demonstrated their utility in differentiating healthy animals from those with late-stage JD, providing potential biomarkers for MAP infection and disease progression.

## Introduction

Eukaryotic gene expression is fine-tuned by hundreds to thousands of short non-coding transcripts known as microRNAs. These single-stranded 21–25nt molecules exert cytoplasmic mRNA cleavage or translational repression but are also deliberately released into extracellular environments, in which they are associated with proteins or enclosed within microvesicles or exosomes^1^. Although the functions of extracellular miRNAs remain somewhat unclear^2^, the consensus that different biological processes give rise to different but specific small RNA signatures envisions their utility as potential prognostic or diagnostic markers^3^. For a range of human maladies, this has mostly been explored in circulatory fluids namely plasma and serum^3–5^, although there is a growing emphasis on other biofluid sources of miRNAs such as cerebrospinal fluid, tears, saliva, bronchoalveolar lavage, sweat, milk, urine, semen and faeces. Using the latter case of faeces as an example, specific miRNAs have been shown to be differentially expressed in faecal samples of colorectal cancer cases relative to those from healthy controls^6^.

In the bovine sphere, faecal miRNAs have not been described but if they were present they would offer a non-invasive prognostic or diagnostic route for pathological conditions of the intestine. One such avenue would be their potential application to Johne’s Disease (JD) in cattle, a progressive granulomatous inflammation of the ileum arising from infection with *Mycobacterium avium* subspecies *paratuberculosis* (MAP). Currently available assays for the diagnosis of MAP infection are based on serological responses and culture of MAP from faecal samples. These tests suffer with issues of sensitivity and specificity, as well as the slow growth rate of MAP (6–8 weeks to form colonies). Novel biomarkers are therefore needed that could augment current control tools, and allow the disease status of cattle infected with MAP to be assessed in terms of progression to clinical disease.

In general, healthy cattle are susceptible to infection through the faecal-oral route but resistant to clinical signs, with a key role for CD4^+^ T lymphocytes producing IFN-γ and TNF-α to prevent mycobacterial dissemination^7^. MAP is not eliminated in all animals, however, and approximately 5–10% of animals will develop a decline in protective Th1 responses within 2–6 years due to production stresses and other factors, facilitating MAP spread throughout the lamina propria and submucosa. At this phase, ineffective Th2 responses arise with the presence of increased MAP shedding in faeces. Thus, there are different phases of MAP infection, each with distinct immunological and pathological characteristics in the intestine. These distinct phases are potentially governed by distinct miRNA profiles; if these miRNA are released extracellularly, their exploitation as biomarkers could provide a novel route to defining the progression of disease in infected animals.

Previous studies in MAP infected cattle have measured miRNA expression in serum^8^, whole blood^9^ and ileal tissues^10^. We have previously investigated circulating miRNA expression in peripheral blood at silent and subclinical MAP infection stages but detected minimal changes that did not provide diagnostic benefit^11,12^. It may, however, be more rewarding to investigate extracellular miRNAs at a local level, with faeces providing a route to explore miRNA species and abundance in the context of the intestinal immune response. Intestinal epithelial cells (IEC) in mice have been shown to be a major source of faecal miRNAs^13^. Furthermore, it is possible that intestinal immune cells may contribute to the abundance of miRNA transcripts, either though active vesicle release or due to necrosis or apoptosis. Particularly in situations where the epithelial barrier has been destabilised, such as with JD, there would be ample opportunity for apical release of miRNAs from the lamina propria. Epithelial damage is rampant at this stage, with uncontrolled inflammation leading to leukocyte influxes, thickening of the intestinal wall and reduction in fluid and nutrient absorption. This is in stark contrast to the intestinal tissue of healthy cattle, where epithelial barrier function is maintained and inflammatory responses are moderated. Hence, intestinal miRNA expression signatures, and release into faeces, could be significantly different between healthy cattle and those with clinical JD. Furthermore, as faecal samples are routinely taken for MAP culture from cattle under JD control programmes, processing of faecal samples for both culture and miRNA abundance would allow faecal miRNA analysis to integrate with existing JD screening protocols without the need for extra sampling.

A question arises over the most appropriate and reliable technology to investigate the expression of multiple miRNAs in faeces. Small RNA sequencing (RNA-seq) has the global capability to identify known and novel miRNome constituents but may lack sensitivity in terms of being able to select mammalian miRNAs over bacterial small RNAs and degraded, short bovine transcripts that are apparent in the faecal environment. Small RNA-seq has been used in human faecal samples and only a small number of miRNAs were identified. As an alternative, quantitative real-time PCR has an advantage over small RNA-seq in terms of sensitivity. Reagents are available to assess high-throughput miRNA expression but few have been customised specifically for bovine small RNAs. Nevertheless, the substantial miRNome sequence identity conservation between *Homo sapiens* and *Bos taurus* suggests theoretical reagent cross-species hybridisation. Such compatibility may also be extended to Nanostring nCounter miRNA Expression Assays, which hybridize paired colour-coded probes to target miRNAs and enable specific and sensitive digital detection.

In this study, we confirm the cross-species applicability of reagents developed for human miRNA quantification with bovine miRNAs, and we utilise this technology to report that approximately 258 distinct miRNAs are detectable in the faeces of cattle. We further identify differential abundance of faecal miRNA between healthy controls and clinical JD cases, suggesting the potential of faecal miRNA as JD diagnostic and/or prognostic biomarkers.

## Methods

### Samples

We performed two independent analyses of faecal miRNA using healthy controls versus cattle with confirmed JD. Biobanked, residual faeces were used (stored at −80 °C) that had originally been collected for MAP faecal culture as part of routine JD diagnostics. We initially selected six clinical cattle across different herds that were dual ELISA positive and faecal positive for MAP. Faecal samples from six cattle from a single herd that had shown no prior signs of MAP infection (consistent ELISA and faecal negativity on a yearly basis) were used as healthy controls (see Table 1 for a breakdown of cattle characteristics). This is referred to as ‘Experiment 1’ in the text.

**Table 1:** Description of samples used in this study.

	Sample ID	Culture and ELISA	Breed	Type	Age (Years)
Experiment 1					
	TB14-004052	Negative	Simmental Cross	Cow	3.13
	TB14-004053	Negative	Simmental Cross	Cow	2.78
	TB14-004054	Negative	Simmental Cross	Cow	2.77
	TB14-004055	Negative	Simmental Cross	Cow	2.77
	TB14-004056	Negative	Simmental Cross	Cow	2.77
	TB14-004059	Negative	Simmental Cross	Cow	2.77
	TB15-000427	Positive	Limousin	Bull	1.79
	TB15-000780	Positive	Holstein	Cow	5.95
	TB15-001260	Positive	Aberdeen Angus Cross	Cow	6.92
	TB15-001405	Positive	Angus	Bull	1.90
	TB15-003631	Positive	Angus	Cow	2.83
	TB15-003925	Positive	Limousin Cross	Steer	2.14
Experiment 2					
	TB18-002207	Positive	Holstein Friesian	Cow	4.10
	TB18-002354	Positive	Limousin Cross	Cow	3.64
	TB18-002355	Positive	Blonde D’Aquitaine Cross	Steer	3.44
	TB18-002415	Positive	Friesian Cross	Cow	6.16
	TB18-002428	Negative	Limousin Cross	Steer	1.18
	TB18-002429	Negative	Simmental Cross	Cow	4.21
	TB18-002430	Negative	Charolais Cross	Heifer	2.25
	TB18-002431	Negative	Simmental Cross	Cow	6.74
	TB18-002499	Positive	Limousin Cross	Heifer	2.45
	TB18-002784	Positive	Holstein Friesian	Cow	3.23
	TB18-002996	Positive	Holstein Friesian	Bull	2.80
	TB18-003103	Positive	Charolais Cross	Cow	10.15

We subsequently included an additional group of faecal samples from biobanked samples that included greater variation in breed and age of animals. This second set of samples aimed to both confirm the reproducibility of results from Experiment 1, and improve our total number of samples when combined to detect miRNA that consistently showed differential abundance across diseased and healthy animals. The second group had seven animals that were dual ELISA and faecal positive for MAP, plus an additional four negative control animals (Table 1). This is referred to as ‘Experiment 2′.

### RNA Extraction

RNA was extracted from 250 mg faeces using the PowerMicrobiome RNA Isolation Kit (MO BIO, Qiagen) according to the manufacturer’s specifications, with nucleic acids eluted in 100 µl of nuclease-free water. Total RNA was subsequently quantified using a NanoDrop-1000 spectrophotometer.

### NanoString detection of miRNAs

NanoString nCounter Human v3 miRNA Expression Assays consisting of 800 targets were initially used with 100 ng of purified bovine RNA from whole blood (collected in Tempus Blood RNA Tubes) sera and endometrium to confirm cross-species compatibility. Human brain RNA served as a positive control. Subsequently, Nanostring nCounter assays were used with 300 ng of faecal RNA to investigate differential miRNA expression between the clinical Johne’s (n = 6) and control groups (n = 6) in Experiment 1. Nanostring nCounter data was analysed using nSolver Analysis Software. For each sample a cut-off of 20 miRNA counts, selected based on the mean of the negative control targets, was applied to minimise false positives.

### cDNA synthesis and quantitative real-time PCR

For the PCR arrays, 40 ng of RNA extracted from faeces was used with the miRCURY LNA cDNA synthesis kit II (Exiqon) in 40 µl reactions according to the manufacturer’s recommendations to polyadenylate and reverse-transcribe miRNAs. The resulting cDNA was subsequently mixed 1:1 with ExiLENT SYBR Green master mix (Exiqon). ROX Reference Dye (Invitrogen) was added at 50 nM to the final mixture. The mixture was used to prepare 10 μl PCR reactions with the ready-to-use miRNome miRNA panels I and II version 4 (Exiqon 384-well plates), which collectively consist of 752 human small RNA targets. Amplification was performed on a QuantStudio 7 Flex Real-Time PCR System (Applied Biosystems) using the following cycling conditions: 95 °C for 10 min followed by 40 amplification cycles at 95 °C for 10 s and 60 °C for 10 s. The ramp-rate was 1.6 °C/s and melting (dissociation) curve analysis was performed on each PCR product. The melt curve profile was used to exclude mismatch PCR product data; only wells that produced a single melt curve peak and showed consistent Tm across all the samples were included in our analyses.

### PCR panel data analysis

#### Experiment 1

As a measure of quality control to reduce unreliable data and potential PCR artefacts, miRNA targets that had aberrant dissociation curves post-amplification were omitted from subsequent analyses. Consequently, data for the remaining miRNAs was processed using Exiqon GenEx software (version 6). Pre-processing of this data sequentially involved inter-plate calibration between panels I and II, removal of samples with cycle threshold (Ct) values>37 and normalization to global mean. Relative miRNA quantities between the clinical JD group and the negative control group were subsequently determined and converted to log_2_ scale.

#### Experiment 2

The same pre-processing methods were used for this group: Ct threshold>37, exclusion of samples with multiple dissociation peaks, inter-plate calibration and normalization to global mean, followed by calculation of the fold change between the clinical JD group and the healthy control group.

### Differential Expression analysis

Samples for both groups were combined together to create a single set of the detectable targets using a Python script. The overlapping detectable genes in both sets were kept for differential expression analysis. Relative abundance was quantified using the dCt method. Since a reference gene was not available we calculated a normalisation factor as the mean Ct value for all miRNAs in each sample. We then subtracted this from the raw Ct value to find the dCt. A ddCt value was then calculated by subtracting these values from the average of the negative samples. These values could then be used directly in a statistical test between groups. The groups were compared using the Mann Whitney non-parametric test for unpaired samples with the Python Scipy package^14^. Any statistically significant miRNAs were manually investigated and only those miRNA that had Ct < 37 in at least 3 animals per group were considered biologically significant; this criterion was used to exclude data points prior to the statistical test for differential expression. We performed these tests independently for both arrays, and subsequently with all samples combined. Only those miRNAs with significance (p-value < 0.05) in both experiments were selected as candidates that showed differential abundance between confirmed JD animals and healthy controls.

### Identification of unreported bovine miRNA orthologs

Basic Local Alignment Search Tool (BLAST) was used to identify close or exact matches between the Exiqon human miRNA panel and known bovine miRNA from miRBase (Release 22). BLAST parameters appropriate for short sequences, i.e. ‘-e 1 -W 7 -S 1’ were used and results with lower than an alignment length of 18 and more than one mismatch were rejected. The sequences of PCR-detected bovine miRNAs that, however, had revealed no reported bovine orthologs in miRBase, were subsequently aligned against the bovine genome (ARS-UCD1.2) via BLAST to probe for exact matches. Possible precursors were then excised from the flanking sequence in both 5′ and 3′ directions at multiple lengths. The secondary structure of each was then scored using a feature classifier and the best structure selected; those with no reliable precursor were rejected. The location of our target mature sequences along the precursor hairpin was then checked for consistency with a true miRNA. We also detected if the precursor was located inside a known gene using the ensembl API. All analysis, processing of BLAST results and novel miRNA prediction was performed using the Python smallrnaseq package^15^ using a similar approach to that described in the miRanalyzer method^16^. The forna (force-directed RNA) web application^17^ was used to visualize miRNA hairpin structures.

## Results

### Detection of miRNAs in bovine faeces using individual PCR assays

miRNAs including miR-223, miR-19b, miR-27b, miR-30d, miR-24 and miR-16 have been shown to be highly expressed in human faeces. Considering the predicted conservation with regard to sequence and function of miRNAs between mammalian species, our first approach was to investigate these six miRNAs for their presence in bovine faeces using the most sensitive means of detection, namely quantitative real-time PCR. Reassuringly, all of these miRNA were detectable in the 12 bovine faecal samples from Experiment 1. Notably, miR-223 and miR-16 had average Ct values <30 indicating that they are abundant in bovine faeces (data not shown). Control reactions lacking reverse-transcriptase were used to confirm that amplifications were not simply a result of residual genomic DNA contamination

### Characterisation of bovine faecal miRNome

We utilised two approaches to identify miRNA in bovine faecal samples. The first of these used the Nanostring nCounter Human v3 miRNA Expression Assays (Nanostring). This hybridization-based assay probes for 800 miRNA targets. Bovine sample types that are known to be rich sources of small RNAs were utilised initially to validate the cross-reactivity of human miRNA probes. Human brain RNA served as a typical positive control as 251 miRNAs were identified (count cut-off>20). 252 transcripts were identified in bovine endometrium and each of the top 20 highest expressed miRNAs identified had approximately three times as many counts as the top 20 brain miRNAs. Albeit to a lesser extent, miRNAs were also detected in bovine whole-blood (86) and serum (26). Seventeen miRNAs including miR-451a, miR-25-3b, miR-150-5p, miR-191-5p and miR-423-5p were found in common between the whole-blood and serum datasets (Table S1). Evidently, a subset of human probes successfully cross-reacted with bovine miRNAs. However, on application to the faecal samples in Experiment 1 there were, on average, only 40 miRNAs detected with variable numbers detected between samples (ranging from 19-62 distinct transcripts). Hence, it was deemed that the Nanostring nCounter Human v3 miRNA would not be sensitive enough to explore the bovine faecal miRNome.

We subsequently moved to a second approach, namely faecal miRNome PCR miRNome arrays (Exiqon) which target 752 different miRNAs. These arrays are designed to target human transcripts; however, a subset of the miRNA sequences are either identical to the bovine orthologs or with single mismatches. Nevertheless, to determine the reliability of this approach, it was first important to assess the quality of the high-throughput PCR data arising from the bovine faecal samples.

**Figure 1:**
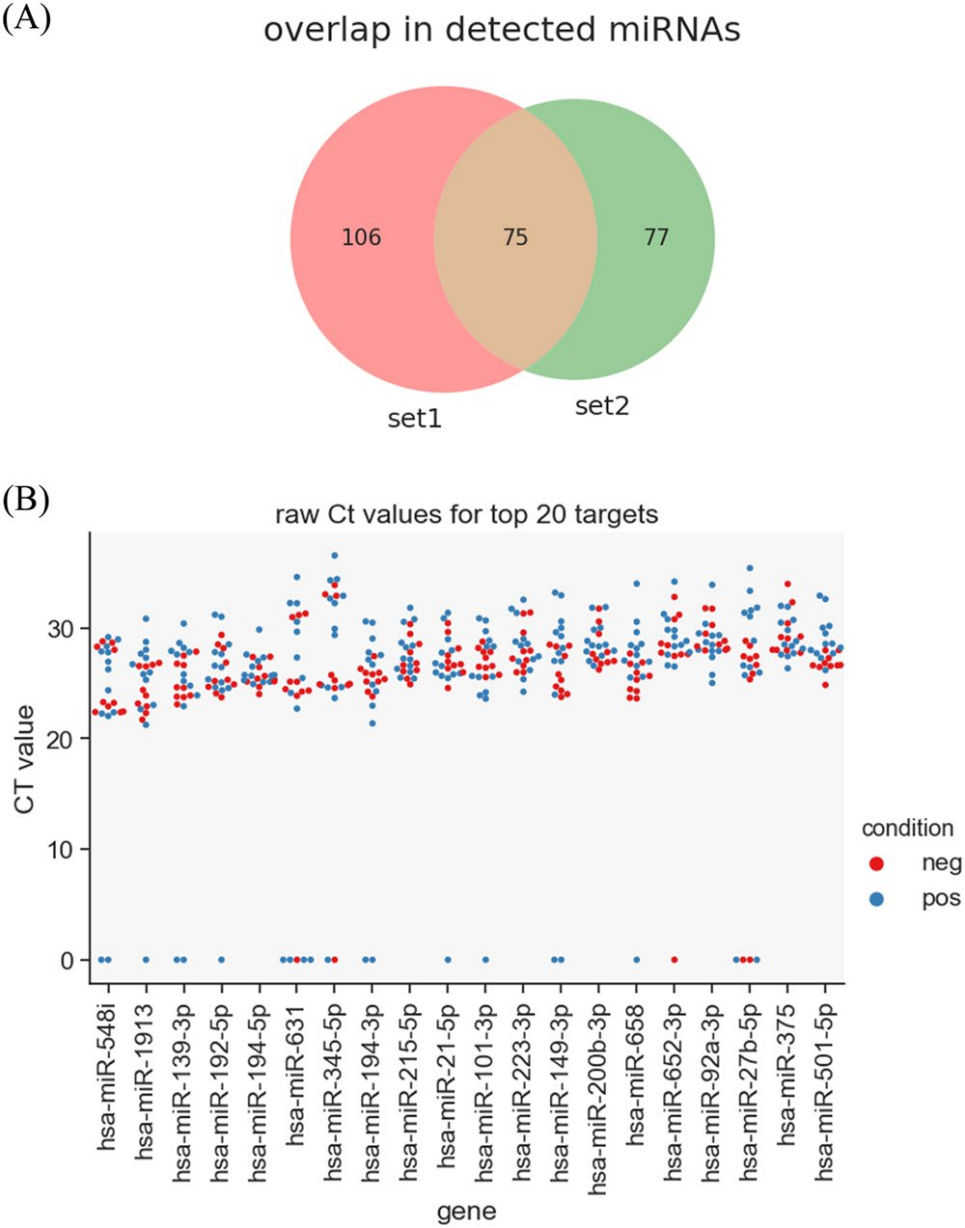
Characterisation of bovine faecal miRNome. (**A**) Overlap between miRNAs detected in both PCR panel sets from experiment 1 (set 1) and experiment 2 (set 2). (**B**) Raw Ct values generated by miRNome PCR panels for the 20 highest expressed miRNAs in bovine faeces (clinical JD – n = 13, negative controls – n = 10). Poor quality samples are shown as zero Ct. There was no significant difference between positive and negative samples.

Initial analysis of Experiment 1 suggested that approximately 689 miRNA targets had been successfully amplified with Ct values <37. However, a closer inspection of the dissociation curves revealed that many PCR products were artefacts or displayed nonspecific amplification. Based on dissociation curve analysis poor quality samples were given empty Ct values. Any target with more than six out of twelve valid Ct values was considered as having high confidence. This filtering resulted in 181 high confidence miRNA targets that remained for subsequent analyses. The same approach was used for filtering the results from Experiment 2, with 160 high confidence targets identified. Of these two experimental data sets, 75 miRNAs overlapped (Fig. 1A). Thus a total of 258 detectable miRNA were found from both sets using the Exiqon human miRNA panels.

Based on average Ct values across all samples, in descending order, the most abundant miRNAs in bovine faeces were miR-548i, miR-1913, miR-139-3p, miR-192-5p, miR-194-5p, miR-631 and miR-345-5p (Fig. 1B). Sample to sample variation was apparent for most miRNAs. No miRNA was stably expressed sufficiently to be used as a reference gene. We therefore used global normalisation for calculating dCt values.

Relative to bovine miRNAs, human miRNAs have been characterised in greater depth, particularly with regard to the number of unique miRNA discovered to date. It was thus possible that some of the 258 amplified miRNAs had not been reported in bovine samples previously. The PCR panel targets were compared against the 1030 known bovine miRNA precursor sequences contained in miRBase (release 22) using BLAST to identify exact or very closely matching cattle orthologs. Collectively, 303 potential targets were found in this search. Of the final 258 human targets actually detected we found 177 that had exact or close matches to a known bovine 5′ or 3′ miRNA mature sequence. Close matches were defined as those targets with single mismatches or 3′ length variants (of any length) with the miRBase sequence. Of these 177 miRNA: 70 were exact matches, 43 length variants and 18 had a single mismatch. The remaining 46 mapped to a miRBase precursor but not the recorded mature sequence. These latter miRNA were checked for complementarity to the recorded mature sequence in each case and some excluded where they did not match the mature sequence; 37 of these were confirmed as being complementary and in the correct position in the precursor. This latter result indicates that these 37 miRNA are likely star sequences not yet recorded in miRBase, and full details are listed in Supplementary Table S2. It is possible that many of new miRNA are rarely present, or in too low abundance, to have been detected previously with sufficient counts in bovine RNA-seq datasets.

### Novel miRNA prediction

**Figure 2:**
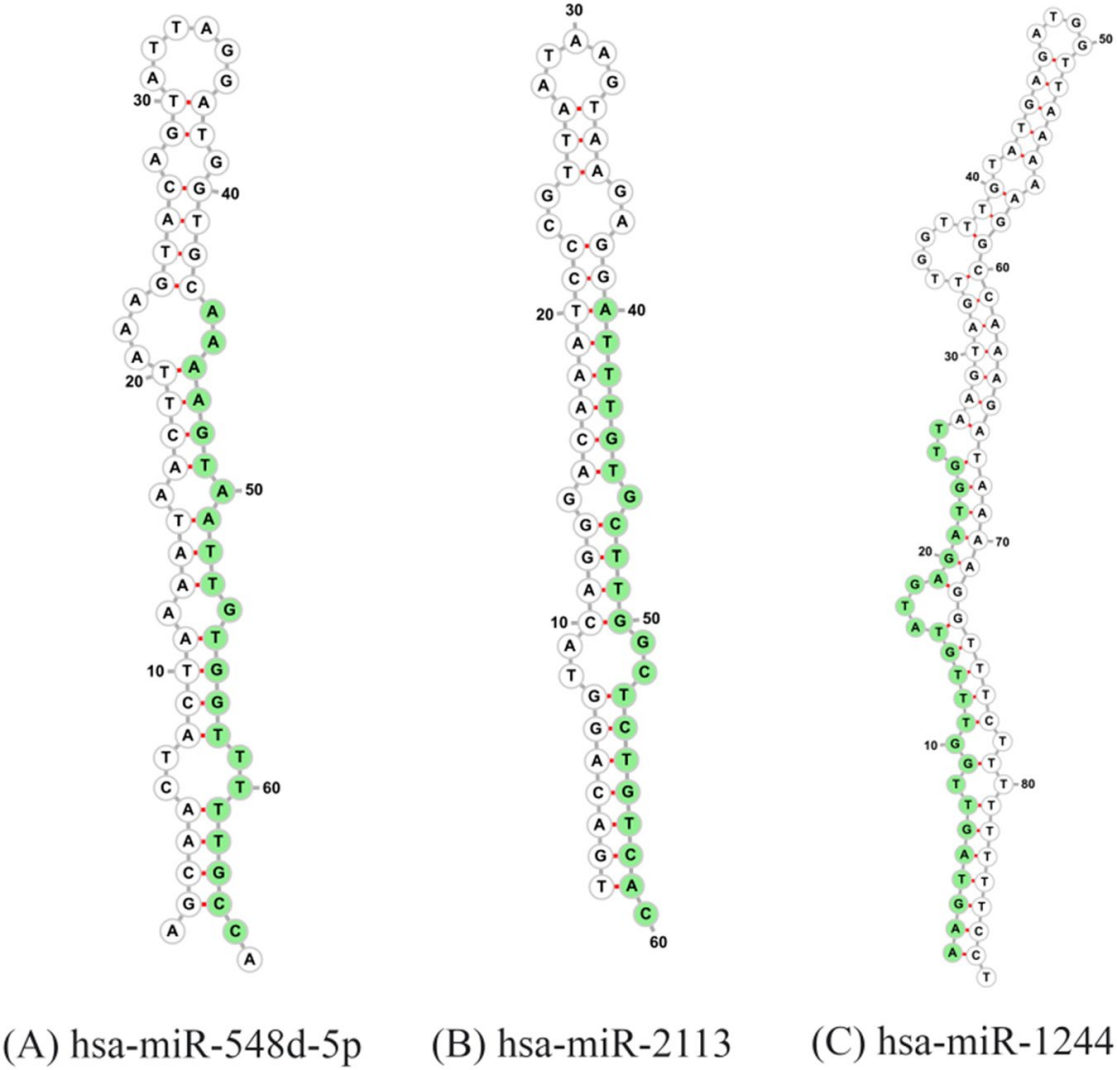
Novel bovine miRNA. Three novel miRNA predicted precursors generated from detected sequences that are currently not present in the bovine miRBase database (Release 22). Mature sequence is shown in green.

From the analysis reported above, there remained 68 miRNA sequences that we could not match closely to any known bovine precursors. Due to the nature of the samples, these could come from multiple sources. To identify them as possible novel bovine miRNAs we excised potential precursors located from a BLAST of the mature sequences to the bovine (ARS-UCD1.2) genome. Sequences surrounding the target location in the genome were folded into multiple possible precursors, scored and then manually reviewed for consistency with the position of the target in the precursor. Three possible candidates from targets hsa-miR-548d-5p, hsa-miR-2113 and hsa-miR-1244 were found (Fig. 2), with the mature sequence shown in green. The precursors are not located inside any known genes. The coordinates and mean free energy of precursors are listed in Table 2.

**Table 2:** Potential novel bovine miRNAs found with precursor co-ordinates in bovine genome.

Human ortholog	Mature sequence	MFE*	Bovine genome coordinates^¶^
hsa-miR-548d-5p	AAAAGTAATTGTGGTTTTTGCC	−11.3	NC_037357.1:11388342..11388406-
hsa-miR-2113	ATTTGTGCTTGGCTCTGTCAC	−26.8	NC_037336.1:51693442..51693504-
hsa-miR-1244	AAGTAGTTGGTTTGTATGAGATGGTT	−12.2	NC_037329.1:47897067..47897155+

*MFE = mean free energy.

^¶^Bovine genome version ARS-UCD1.2.

### Identification of differential miRNA abundance in animals with Johne’s Disease

**Figure 3:**
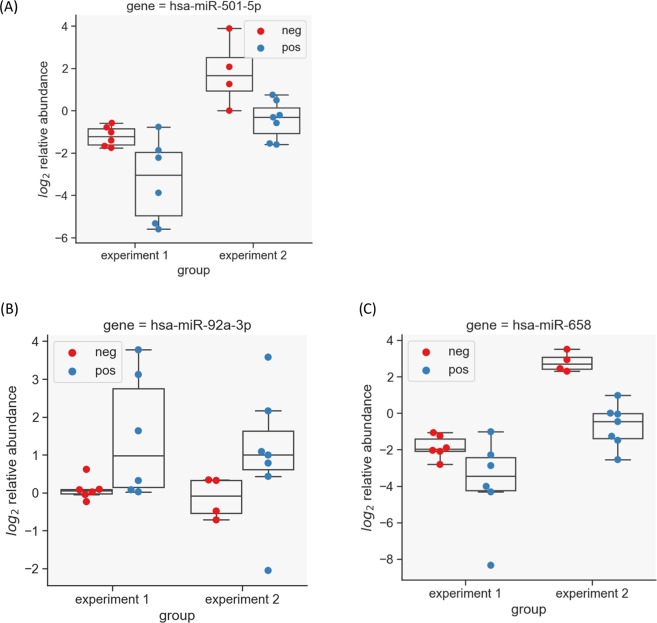
miRNA markers of Johne’s Disease (JD). Differential faecal miRNA expression in response to clinical JD shows three significant miRNAs in experiments 1 and 2. ‘Positive’ (blue dots) are animals with confirmed JD, while ‘negative’ (red dots) are healthy controls. The y-axis shows log_2_ relative abundance calculated by taking the negative ddCt values to indicate fold change. (**A**) hsa-miR-501-5p is present in lower abundance in faeces from animals with confirmed JD relative to controls. (**B**) hsa-miR-92a-3p is more abundant in faeces from JD positive animals compared to controls. (**C**) hsa-miR-658 has lower abundance in faeces from animals with JD.

Differential expression analysis was done to find changes in the abundance of miRNA in animals confirmed as MAP ELISA-positive/faecal-positive vs healthy controls. This was done on both sets independently and then for the entire set of 75 overlapping miRNAs. Only miRNAs found to be significant in all cases were considered as true positives. A non-parametric unpaired test was applied to the normalised dCt values. For Experiment 1, 12 miRNA transcripts were found to have significantly differential abundance; in experiment 2, seven miRNA showed differential abundance (Table 3). Three miRNA showed the same trend in abundance across both experiments; hsa-miR-92a-3p was found to be more abundant in samples from animals with confirmed JD, while miR-501-5p and hsa-miR-658 showed lower abundance in clinical samples versus controls (Fig. 3). Only hsa-miR-92a-3p has a known bovine orthologue, namely bta-mir-92a-1.

**Table 3:** miRNA found to be differentially expressed in either Expt 1 or 2, or both.

miRNA name	Expt 1*	Expt 2	Bovine ortholog^¶^	Sequence
hsa-miR-149-3p	+	−	bta-miR-149-3p	AGGGAGGGACGGGGGCTGTGC
hsa-miR-658	+	+		GGCGGAGGGAAGTAGGTCCGTTGGT
hsa-miR-92a-3p	+	+	bta-miR-92a	TATTGCACTTGTCCCGGCCTGT
hsa-miR-375	+	−	bta-miR-375	TTTGTTCGTTCGGCTCGCGTGA
hsa-miR-501-5p	+	+		AATCCTTTGTCCCTGGGTGAGA
hsa-miR-1258	+	−		AGTTAGGATTAGGTCGTGGAA
hsa-miR-222-3p	+	−	bta-miR-222	AGCTACATCTGGCTACTGGGT
hsa-miR-320b	+	−	bta-miR-320b	AAAAGCTGGGTTGAGAGGGCAA
hsa-miR-20a-5p	+	−	bta-miR-20a	TAAAGTGCTTATAGTGCAGGTAG
hsa-miR-27b-3p	+	−	bta-miR-27b	TTCACAGTGGCTAAGTTCTGC
hsa-miR-106b-5p	+	−	bta-miR-106b	TAAAGTGCTGACAGTGCAGAT
hsa-miR-29c-3p	+	−	bta-miR-29c	TAGCACCATTTGAAATCGGTTA
hsa-miR-652-3p	−	+	bta-miR-652	AATGGCGCCACTAGGGTTGTG
hsa-miR-320a	−	+	bta-miR-320a	AAAAGCTGGGTTGAGAGGGCGA
hsa-miR-151a-5p	−	+	bta-miR-151-5p	TCGAGGAGCTCACAGTCTAGT
hsa-miR-15b-3p	−	+		CGAATCATTATTTGCTGCTCTA

*‘+’ = detected; ‘−’ not detected; miRNA detected across both experiments in bold.

^¶^Bovine orthologs are indicated that are exact matches to the human sequence.

Testing the miRNAs for significant differential abundance in experiment 1 and 2 individually, or with all 24 samples from both experiments combined, hsa-miR-92a-3p and hsa-miR-658 showed statistically significant differential abundance across all cases; hsa-miR-501-5p was outside the level of statistical significance when tested in this combined analysis (p-value of 0.06).

## Discussion

A number of groups have previously described the differential expression of miRNA between MAP infected and control groups of cattle across a range of biological samples. Using miRNA-seq of RNA from whole blood of infected, exposed and negative cattle, Malvisi *et al*.^9^ identified 9 DE miRNA between negative and infected groups. Using an ileal loop model in neonate calves, Liang *et al*.^10^ identified 9 miRNAs that showed differential expression between infected and control tissues. At the level of serum, Gupta *et al*.^8^ used Nanostring human miRNA panels to detect 26 miRNA in bovine serum. Quantifying miRNA abundance in serum from cattle stratified based on JD presentation (control, mild, moderate, severe) they revealed that a model employing the expression of just 4 miRNA could separate moderate and severely infected animals from the control and mild animals. Conversely, in our previous work using samples from experimental MAP infection models, no significant differential abundance in circulating miRNA could be detected using RNA-seq when comparing uninfected to early stage MAP infection^11^, or from early vs late stage MAP infection^12^.

In addition to the presence of miRNAs in tissue and blood, miRNAs produced in response to pathological changes triggered by MAP infection in the intestine and shed into the lumen and faeces may represent an untapped novel diagnostic, and potentially prognostic, tool to monitor infection and disease status in cattle. In this work, by initially investigating bovine orthologs of miRNAs shown to be expressed in human faeces (miR-223, miR-19b, miR-27b, miR-30d, miR-24 and miR-16), we detecetd miRNA amplification signals using RNA from a low quantity of bovine faeces. Thus, this was evidence that miRNAs are present in bovine faeces and that they are detectable using reagents developed for human homologs.

A global strategy was consequently pursued to firstly characterise the faecal miRNome and to then explore the potential of faecal miRNA as biomarkers for disease status in cattle with confirmed JD. We deemed human miRNome arrays a valuable exploratory tool as the 752 targets present on these arrays are among the best characterised miRNAs in the human field. A large portion of these miRNA targets led to spurious amplification products in RNA extracted from bovine faecal samples, with many primer-dimers or non-specific amplification products based on dissociation curve analysis. This left 258 “high confidence” bovine miRNAs that could be detected in faecal samples across both experiments. Many well characterised miRNAs were represented in this set, but 3 of the amplified targets had not been previously reported in bovine samples.

Three miRNAs displayed significant differential abundance in faeces samples from cattle with JD, and the human orthologs of these transcripts are known to be expressed in intestinal tissue^18^. Changes in ileal miRNA release into the lumen as a result of JD pathology may account for the differential faecal miRNA expression, but we are aware that the specificity of these miRNAs for JD will need to be tested against other intestinal diseases in cattle with inflammatory pathology. Indeed, while several studies have shown that faecal miRNAs are differentially expressed in response to colorectal cancer in humans^19^, studies on miRNA changes in response to inflammatory intestinal condition in humans, such as ulcerative colitis, are more limited^20^.

Some caveats with the work should be noted. For example, it has been shown by others that different RNA isolation procedures can affect both the quantity and quality of miRNAs extracted from substrates^21–23^. Hence, further optimisation of the methods employed in this work may allow a greater repertoire of miRNA to be detected that may, in turn, improve discrimination of JD-affected from healthy cattle. In addition, greater sample numbers may have allowed more robust inferences on miRNA abundance across diseased and healthy animals to be made. While we only detected three differential miRNAs after our stringent filters were applied, we emphasize that we were using reagents optimised for the detection of human miRNA; hence, it is entirely plausible that future work using targeted bovine miRNA panels will detect greater variation in the miRNA signal between control and JD states.

Finally, the question remains as to whether the miRNA markers identified here from cattle in the clinical-phase of disease may have utility as prognostic biomarkers to monitor disease progression in subclinical phases of MAP infection. Clearly, the fact that faeces are regularly used for MAP culture from suspect JD animals makes the parallel examination of faeces for miRNA an attractive adjunct to culture for JD diagnostics. Future work will seek to address the value of faecal miRNA biomarkers to ultimately improve JD control.

## Supplementary information

Supplementary Information.
