# Supplementary material for: Identification of microRNAs in bovine faeces and their potential as biomarkers of Johne’s Disease
Source: Sci Rep. 2020 Apr 3;10:5908. doi: 10.1038/s41598-020-62843-w (PMC7125074; doi:10.1038/s41598-020-62843-w)
Supplement: Supplementary file 1 — Supplementary Information. [file 41598_2020_62843_MOESM1_ESM.pdf]

Identification of microRNAs in bovine faeces and their potential as biomarkers of Johne's Disease.

Ronan G. Shaughnessy, Damien Farrell, Bojan Stojkovic, John A. Browne, Kevin Kenny and Stephen V. Gordon

Supplementary Information:
Table S1
Table S2

Supplementary Information

Table S1: Results using Nanostring nCounter Human v3 miRNA assays with bovine faecal RNA

Human brain counts (control)		Bovine endometrium counts		Bovine whole blood counts		Bovine serum counts	
let-7a-5p	44377	let-7a-5p	139491	miR-451a	95796	miR-4454	844
miR-125b-5p	29776	let-7b-5p	95134	let-7b-5p	11307	miR-1246	422
miR-9-5p	27650	miR-125b-5p	81239	miR-25-3p	5884	miR-150-5p	146
let-7b-5p	20407	miR-199a/b-3p	61079	miR-144-3p	3861	miR-4516	137
miR-29b-3p	12267	miR-23a-3p	29267	let-7a-5p	3732	miR-423-5p	126
miR-181a-5p	9681	miR-126-3p	25039	miR-150-5p	3097	miR-451a	117
miR-451a	8278	miR-200b-3p	21965	miR-181a-5p	2663	miR-223-3p	115
let-7c-5p	4666	miR-199b-5p	20775	miR-191-5p	2593	miR-23a-3p	99
let-7g-5p	4441	miR-148a-3p	16078	miR-15b-5p	2157	miR-191-5p	77
let-7d-5p	3872	miR-145-5p	12300	let-7d-5p	1379	miR-1915-3p	50
miR-125a-5p	3538	let-7g-5p	11860	miR-423-5p	1061	miR-4532	43
miR-26a-5p	3388	let-7c-5p	10831	miR-15a-5p	1006	miR-3065-5p	42
miR-126-3p	3105	miR-200a-3p	10140	miR-93-5p	905	miR-122-5p	39
miR-124-3p	2950	miR-199a-5p	9877	miR-1306-5p	899	miR-630	39
let-7i-5p	2377	miR-99a-5p	9684	miR-142-3p	799	miR-181a-5p	35
miR-218-5p	2375	miR-23b-3p	9133	miR-16-5p	541	miR-575	33
miR-342-3p	2343	miR-21-5p	8720	let-7c-5p	414	miR-3195	30
miR-219a-2-3p	2157	let-7i-5p	8703	let-7g-5p	402	miR-25-3p	27
let-7e-5p	2013	miR-143-3p	8128	let-7i-5p	398	miR-1290	26
miR-99a-5p	1950	miR-10b-5p	7848	miR-92a-3p	271	miR-873-3p	26

Table S2: Newly detected bovine mature sequences with the human targets used to detect them.

Target	Sequence	Start*	End	miRBase ID#	Length
hsa-let-7a-2-3p	CTGTACAGCCTCCTAGCTTTCC	50	71	bta-let-7a-2	22
hsa-let-7b-5p	TGAGGTAGTAGGTTGTGTGGTT	81	60	bta-mir-3596	22
hsa-let-7d-3p	CTATACGACCTGCTGCCTTTCT	62	83	bta-let-7d	22
hsa-miR-101-5p	CAGTTATCACAGTGCTGATGCT	15	36	bta-mir-101-1	22
hsa-miR-10b-3p	ACAGATTCGATTCTAGGGGAAT	59	80	bta-mir-10b	22
hsa-miR-130b-5p	ACTCTTTCCCTGTTGCACTAC	13	33	bta-mir-130b	21
hsa-miR-132-5p	ACCGTGGCTTTCGATTGTTACT	23	44	bta-mir-132	22
hsa-miR-138-1-3p	GCTACTTCACAACACCAGGGCC	61	80	bta-mir-138-1	20
hsa-miR-139-3p	TGGAGACGCGGCCCTGTTGGAGT	43	65	bta-mir-139	23
hsa-miR-142-3p	TGTAGTGTTTCCTACTTTATGGA	52	74	bta-mir-142	23
hsa-miR-144-5p	GGATATCATCATATACTGTAAG	13	34	bta-mir-144	22
hsa-miR-148b-5p	AAGTTCTGTTATACACTCAGGC	16	37	bta-mir-148b	22
hsa-miR-15a-3p	CAGGCCATATTGTGCTGCCTCA	51	72	bta-mir-15a	22
hsa-miR-15b-3p	CGAATCATTATTTGCTGCTCTA	58	79	bta-mir-15b	22
hsa-miR-194-3p	CCAGTGGGGCTGCTGTTATCTG	50	71	bta-mir-194-2	22
hsa-miR-194-5p	TGTAACAGCAACTCCATGTGGA	53	32	bta-mir-194b-2	22
hsa-miR-200a-5p	CATCTTACCGGACAGTGCTGGA	14	35	bta-mir-200a	22
hsa-miR-22-3p	AAGCTGCCAGTTGAAGAACTGT	31	10	bta-mir-3600	22
hsa-miR-22-5p	AGTTCTTCAGTGGCAAGCTTTA	69	48	bta-mir-3600	22
hsa-miR-221-5p	ACCTGGCATACAATGTAGATTT	25	46	bta-mir-221	22
hsa-miR-24-3p	TGGCTCAGTTCAGCAGGAACAG	44	65	bta-mir-24-1	22
hsa-miR-26a-2-3p	CCTATTCTTGATTACTTGTTTC	52	73	bta-mir-26a-2	22
hsa-miR-26a-5p	TTCAAGTAATCCAGGATAGGCT	75	54	bta-mir-26c	22
hsa-miR-27b-5p	AGAGCTTAGCTGATTGGTGAAC	19	40	bta-mir-27b	22
hsa-miR-29a-5p	ACTGATTTCTTTTGGTGTTCAG	4	25	bta-mir-29a	22
hsa-miR-324-3p	CCCACTGCCCCAGGTGCTGCTGG	52	74	bta-mir-324	23

hsa-miR-340-5p	TTATAAAGCAATGAGACTGATT	16	37	bta-mir-340	22
hsa-miR-34a-3p	CAATCAGCAAGTATACTGCCCT	65	86	bta-mir-34a	22
hsa-miR-34c-3p	AATCACTAACCACACGGCCAGG	46	67	bta-mir-34c	22
hsa-miR-361-3p	TCCCCCAGGTGTGATTCTGATTT	44	66	bta-mir-361	23
hsa-miR-371a-5p	ACTCAAACTGTGGGGGCACT	12	31	bta-mir-371	20
hsa-miR-378a-5p	CTCCTGACTCCAGGTCCTGTGT	5	26	bta-mir-378-1	22
hsa-miR-411-3p	TATGTAACACGGTCCACTAACC	51	72	bta-mir-411a	22
hsa-miR-505-5p	GGGAGCCAGGAAGTATTGATGT	20	41	bta-mir-505	22
hsa-miR-99b-3p	CAAGCTCGTGTCTGTGGGTCCG	45	66	bta-mir-99b	22
hsa-miR-551b-5p	GAAATCAAGCGTGGGTGAGACC	22	43	bta-mir-551b	22
hsa-miR-615-3p	TCCGAGCCTGGGTCTCCCTCTT	61	81	bta-mir-615	21

*start and end indicate the position inside the precursor.
#miRBase id is the precursor id.
